# Occurrence of Aflatoxin M1 in Three Types of Milk from Xinjiang, China, and the Risk of Exposure for Milk Consumers in Different Age-Sex Groups

**DOI:** 10.3390/foods11233922

**Published:** 2022-12-05

**Authors:** Jianglin Xiong, Fangyuan Chen, Jie Zhang, Weiping Ao, Xiaoling Zhou, Hua Yang, Zhongyuan Wu, Lingying Wu, Chong Wang, Yinsheng Qiu

**Affiliations:** 1 Hubei Key Laboratory of Animal Nutrition and Feed Science, School of Animal Science and Nutrition Engineering, Wuhan Polytechnic University, Wuhan 430023, China; 2 College of Animal Science and Technology, Tarim University, Alaer 843300, China; 3 School of Mathematics and Computer Science, Wuhan Polytechnic University, Wuhan 430023, China; 4 College of Animal Science and Technology & College of Veterinary Medicine, Zhejiang A & F University, Hangzhou 311300, China

**Keywords:** aflatoxin M1, China, milk, occurrence, risk, Xinjiang

## Abstract

Aflatoxin M1 (AFM1), a group 1 carcinogen, is a risk factor to be monitored in milk. This study aimed to investigate the occurrence of AFM1 in milk in Xinjiang, China, and to assess the risk of exposure for milk consumers in different age-sex groups. A total of 259 milk samples including pasteurized milk (93 samples), extended-shelf-life (ESL) milk (96), and raw donkey milk (70) were collected in Xinjiang from January to March in 2022. The AFM1 content of the milk samples was detected using a validated ELISA method. Of the 259 total samples analyzed for AFM1, 84 (32.4%) samples were contaminated at levels greater than the detection limit of 5 ng/L, with the maximum level of 16.5 ng/L. The positive rates of AFM1 in pasteurized milk and ESL milk were 43.0% (*n* = 40) and 45.8% (*n* = 44), respectively, and AFM1 was undetectable in donkey milk. The estimated daily intakes of AFM1 in each age group were lower than the hazard limits and were similar between male and female milk consumers. Therefore, the AFM1 contamination of milk in Xinjiang is low but still needs to be continuously monitored considering that children are susceptible to AFM1.

## 1. Introduction

Aflatoxins are a group of toxic secondary metabolites produced by species of the *Aspergillus* section *Flavi* [1], which have been found to colonize a range of crops including corn and peanuts and their products. Of the natural aflatoxins, aflatoxin B1 (AFB1) is the most common and potent carcinogen [2]. After a lactating animal ingests AFB1-contaminated feed, AFB1 is hydroxylated to aflatoxin M1 (AFM1) in the liver and then secreted into milk, causing AFM1 contamination of related dairy products. In view of the carcinogenicity of AFB1 and AFM1, these two aflatoxins were listed as class I human carcinogens by the International Agency for Research on Cancer (IARC) in 1993 and 2002, respectively [2,3].

To reduce the risk of milk AFM1 intake, more than 60 countries have regulated the maximum levels (MLs) for AFM1 in milk and dairy products to within the range of 0–1000 ng/L [4], including the MLs of 50 ng/L set by the European Commission [5] and 500 ng/L established by China [6] and the United States [7]. In addition, physical, chemical, and biological methods have been used to reduce AFB1 content in feed, reduce the absorption of AFB1 in animal intestine, and detoxify milk AFM1 [8,9]. However, milk and dairy products are still widely contaminated with AFM1 to varying degrees [9], which is a global human health concern. Thus, it is necessary to continuously monitor AFM1 in milk.

Xinjiang Uygur autonomous region (Xinjiang for short), adjacent to Central Asia and located in the northwest of China, is an ethnic minority area, and milk is an important food for its local residents. As a traditional dairy-producing area in China, Xinjiang has a milk output of 2 million tons and an annual milk consumption of 18.72 kg per capita in 2020 [10]. In recent years, the Xinjiang government has issued a series of documents on an action plan for revitalizing the Xinjiang dairy industry, which intends to increase production by 1 million tons of milk through an additional 200,000 Holstein cows raised until 2025 [11]. In addition, Xinjiang’s dairy donkey breeding industry has been supported by local governments in recent years, and the donkey milk produced is usually used to make donkey milk powder [12]. Dairy quality and safety are important guarantees for the revitalization of the dairy industry. Of the many issues of dairy safety, milk AFM1 has attracted much attention from the government, researchers, and citizens because of its strong carcinogenicity. However, little information is available on the risk of exposure to AFM1 among milk consumers in Xinjiang, although it is the main area for milk production and consumption. Moreover, the daily milk consumption of children aged 2–4 years was recommended to be increased from 300 mL to 500 mL [13], which may also increase the intake of milk AFM1. Therefore, we (1) investigated the AFM1 occurrence in the main milk products, pasteurized milk and extended-shelf-life (ESL) milk, and in donkey milk (a common local milk) in Xinjiang, and then (2) assessed the risk of AFM1 exposure for milk consumers in different age-sex groups.

## 2. Materials and Methods

### 2.1. Sample Collection

A total of 259 milk samples, including 93 pasteurized milk samples, 96 ESL milk samples, and 70 raw donkey milk samples, were collected in Alaer City (in southern Xinjiang) and Urumqi City (in northern Xinjiang) of Xinjiang Uyghur Autonomous Region from January to March in 2022. The milk products were collected from supermarkets, convenience stores, and milk stations. The packages of the milk products showed that the collected milk was produced on different dates between January and March. The raw donkey milk samples were collected from milk tanks on different dates. About 30 mL of milk for each sample was collected in a separate sample tube, mixed with preservatives (Broad Spectrum Microtabs II, Advanced Instruments, Norwood, MA, USA), numbered, and stored at −20 °C until analysis.

### 2.2. AFM1 Determination

The AFM1 in the milk samples was detected through the ELISA method using Ridascreen^®^ AFM1 test kit (R1121, R-Biopharm AG, Darmstadt, Germany). The kit has the following features: detection limit of 5 ng/L; cross-reactivity of AFM1 at 100% and AFM2 < 10%. The pretreatment and detection steps of the milk samples were carried out according to our previous study [14]. Briefly, the main detection steps of the kit include: pretreatment of samples, fabrication of antibody-coated plates, sample loading, addition of enzyme conjugate, addition of substrate and chromogen, addition of stop solution, and determination of absorbance.

The absorbance was analyzed using a SpectraMax i3x reader (Molecular Devices (Shanghai) Inc., Shanghai, China) at 450 nm. Milk AFM1 was calculated by RIDASOFT^®^ Win.NET software (Z9999, R-Biopharm AG, Darmstadt, Germany) based on a regression relationship between absorbance and AFM1 concentration. The kit contained AFM1 standards including six concentrations of 0, 5, 10, 20, 40, 50, and 80 ng/L, which were used to establish the regression relationship by the software. To ensure the accuracy of the test results, we verified the standard test results were consistent with the quality control report of the kit for each test. The milk samples were spiked with AFM1 to obtain AFM1-contaminated milk with the levels of 10, 20, and 40 ng/L, which was repeated 3 times, and then these milk samples were analyzed to assess the kit performance. The test showed the following characteristics: a recovery rate of 98.4–108.6% and standard deviation of 1.5–5.3%, which meet the performance parameters set by the Chinese authorities [15].

### 2.3. Exposure Risk Assessment for Different Age Groups

The estimated daily intake (EDI), hazard quotient (HQ), and margin of exposure (MOE) values of AFM1 were used to assess the exposure risk of milk consumers in different age groups. The EDI value was calculated through a deterministic formula [16] by considering the milk AFM1 concentration (ng/mL), the daily milk consumption (mL), and the mean body weight (bw; kg). Thus, our calculation formula was as follows: EDI (ng/kg bw/day) = [(AFM1 content) × (daily milk consumption)]/(bw). To calculate EDI values, the AFM1 content was considered as one of the mean, 75% percentile, and 95% percentile concentrations, which were calculated on the condition that the concentrations in AFM1-negative milk samples were considered to contain one half of the detection limit of 5 ng/L. Moreover, in this formula, data on the body weight of milk consumers were derived from the physical fitness survey of Chinese residents [17]. Data on the daily milk consumption came from a survey of dietary and nutrient intake data [18].

To further assess the risk of AFM1 exposure, the HQ value was adopted and calculated as follows: %HQ = EDI/tolerable daily intake (TDI) × 100, where the TDI value was set as 0.2 ng/kg bw/day. A risk limit of AFM1 was declared by Kuiper-Goodman (1990) [19], which was used in previous studies [14,20,21]. Therefore, an HQ value greater than 100% means that it poses a risk of toxicity.

As another method of assessing the risk of exposure to AFM1, the MOE is calculated as follows: MOE = (570 ng/kg bw/day)/EDI, where the value of 570 ng/kg bw/day is the reference dose, based on the results of an AFM1 exposure test on Fischer rats [22]. If the calculated MOE value is greater than or equal to 10,000, milk AFM1 poses little health risk, while if the MOE value is less than 10,000, it poses a potential health risk.

### 2.4. Statistical Analysis

A difference in the AFM1 levels between pasteurized milk and ESL milk was statistically analyzed with Mann–Whitney nonparametric tests in IBM SPSS Statistics 19.0 (IBM SPSS Inc., Chicago, IL, USA). The variation charts of the HQs and MOEs were made by IBM SPSS software. Statistical significance was established at *p* < 0.05.

## 3. Results

### 3.1. Occurrence of AFM1 in Milk Samples

In this study, a total of 259 milk samples were analyzed for AFM1, with 84 (32.4%) samples at levels greater than the detection limit of 5 ng/L (Table 1 and Table S1). Of the 189 cow milk samples, 84 (44.4%) were positive for AFM1 with a mean concentration of 7.6 ng/L and maximum content of 16.5 ng/L. For pasteurized milk, out of the total 93 samples, 40 (43.0%) were AFM1-positive, with a maximum level of 11.3 ng/L and a mean value of 7.4 ng/L. For ESL milk, of the total 96 samples, 44 (45.8%) were positive for AFM1; the maximum contamination was 16.5 ng/L and the mean value was 7.8 ng/L. No statistical difference (*p* > 0.05) was found in AFM1 content between pasteurized milk and ESL milk. Notably, in donkey milk, of the total 70 samples analyzed, none contained AFM1 above the detection limit of 5 ng/L. The AFM1 content of the cow milk and donkey milk samples tested was neither higher than the ML in China nor higher than that in the European Union (EU).

### 3.2. Risk Assessment of Exposure in Different Age-Sex Groups

EDI was assessed from the AFM1 exposure for milk consumers in the different age-sex groups (Table 2). The ranges of the mean, 75% percentile, and 95% percentile values of EDI in the ten age groups were 0.008–0.053, 0.011–0.078, and 0.017–0.114 ng/kg bw/d, respectively, less than the risk limit of 0.2 ng/kg bw/d reported by Kuiper-Goodman (1990) [19] and in line with those of male milk consumers. The above values of EDI in female consumers were 0.009–0.050, 0.013–0.073, and 0.019–0.108 ng/kg bw/d, respectively. The highest EDI values were found in children aged 2 to 4 years, with male vs. female values of 0.053 vs. 0.050, 0.078 vs. 0.073, and 0.114 vs. 0.108 ng/kg bw/d for the mean and 75% and 95% percentiles, respectively. Moreover, the lowest EDI values (including the mean, 75% percentile, and 95% percentile) were found in adults aged 30–40 years; they were below 10% of the risk limit of 0.2 ng/kg bw/d.

**Figure 1:**
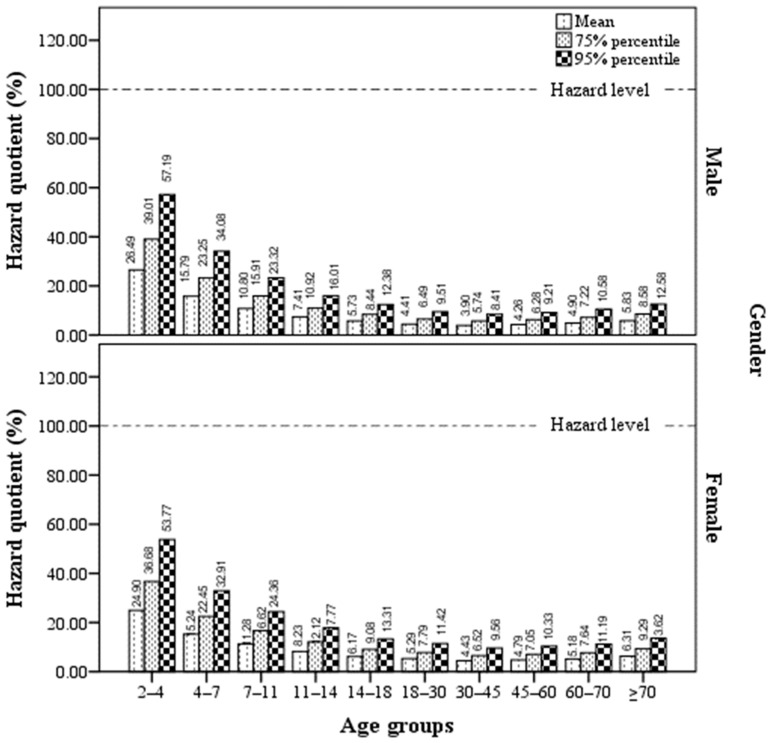
Variation in the hazard quotient (HQ) values of AFM1 exposure for milk consumers in different age-sex groups. The HQs of AFM1 for each age group were lower than the hazard limits, with the highest HQ of AFM1 in children (2–4 years of age) and the lowest in adults (30–40 years of age). The HQs were similar between ten age groups of male and female milk consumers. Note: (1) To calculate estimated daily intake (EDI), the concentrations of AFM1-negative milk samples were assumed to be 2.5 ng/L (half of the detection limit of 5 ng/L), from which the mean, 75% percentile, and 95% percentile concentrations were calculated to be 4.78, 7.04, and 10.32 ng/L, respectively. These concentrations were then used to calculate the corresponding EDI values. (2) %HQ = EDI/tolerable daily intake (TDI) × 100, where the TDI value was set as 0.2 ng/kg bw/day as shown by Kuiper-Goodman (1990) [19]. (3) Milk consumers with HQ values higher than 100% are at health risk.

**Figure 2:**
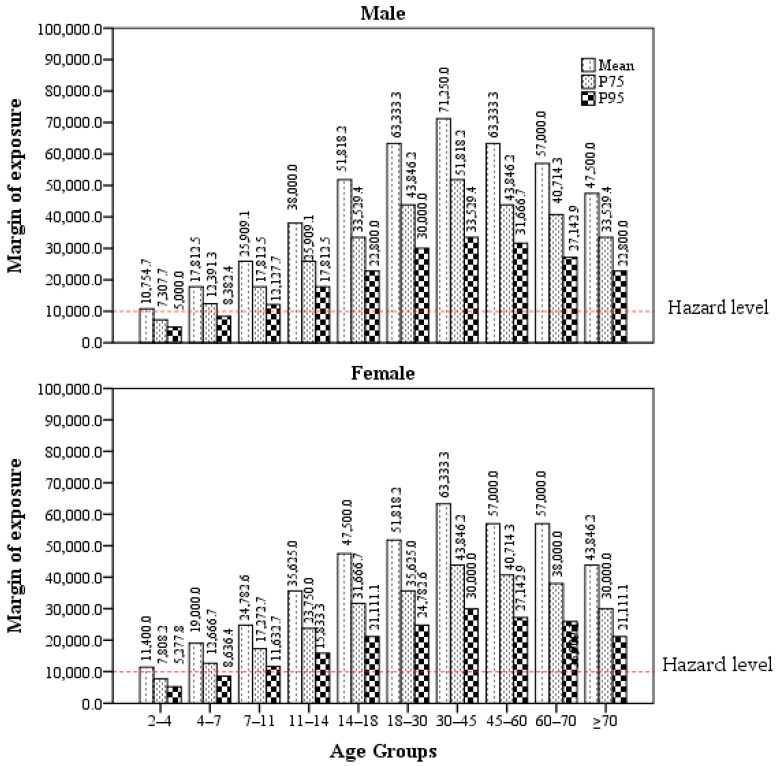
Variation in the margin of exposure (MOE) values of AFM1 exposure for milk consumers in different age-sex groups. The means of the MOEs for each age group were lower than 10,000, indicating that milk consumers were at low health risk overall, while some MOEs, including the 75% and 95% percentiles of MOE values for children aged 2–4 years and the 95% percentile of MOE values for children aged 4–7 years, were below the 10,000, indicating that milk consumers were at health risk to some extent. Note: (1) To calculate estimated daily intake (EDI), the concentrations of AFM1-negative milk samples were assumed to be 2.5 ng/L (half of the detection limit of 5 ng/L), from which the mean, 75% percentile, and 95% percentile concentrations were calculated to be 4.78, 7.04, and 10.32 ng/L, respectively. These concentrations were then used to calculate the corresponding EDI values. (2) MOE = (570 ng/kg bw/day)/EDI, where the value of 570 ng/kg bw/day is the reference dose, based on the results of an AFM1 exposure test on Fischer rats [22]. (3) If the calculated MOE value is greater than or equal to 10,000, milk AFM1 poses little health risk, while if the MOE values is less than 10,000, it poses a potential health risk.

For further analysis, the HQs and MOEs of different age-sex groups were analyzed and plotted, as shown in Figure 1 and Figure 2. Among the 10 different age groups, milk consumers in the 30–45 age group had the lowest HQ. The HQs gradually decreased with age for milk consumers before this age group, whereas they increased with age for milk consumers after this age group. We observed a similar trend of HQs in male and female milk consumers, and the HQs were similar between ten different age groups of male and female milk consumers.

Notably, although the HQ values for each age group showed that milk consumers were at low risk, some MOE values, including the 75% and 95% percentiles of MOE values for children aged 2–4 years and the 95% percentile of MOE values for children aged 4–7 years, were within the risk range, indicating that milk consumers were at health risk to some extent. Overall, the trend of MOE and HQ values were consistent in all age-sex groups.

## 4. Discussion

### 4.1. Occurrence of AFM1 in Pasteurized and ESL Milk

Previous studies have reported the occurrence of AFM1 in pasteurized milk and ESL milk from central, eastern, southern, and northern regions of China from 2013 to 2022 (Table 3). These studies indicated that the AFM1 content of all milk samples was far below the China ML, which is consistent with this study. This indicates that Chinese dairy farms, dairy production enterprises, and Chinese quality supervision departments have strictly prevented and monitored the milk AFM1 contamination. However, the percentage of positive, mean, and maximum concentrations of AFM1 in milk were higher in previous studies (Table 3) than in this study.

Consistent with the findings of this study, no ESL sample was found to have higher AFM1 content than the EU ML in the previous study [25], whereas 0.8–65.4% of the pasteurized and ESL milk had AFM1 exceeding this ML in other studies (Table 3), and greater than zero in the current study. The milk AFM1 contamination in China measured in previous studies is higher than that in the present study, which may be related to the great difference in climate in the sampling areas, considering the influence of climate change on AFB1 production [24,25]. Unlike most of the locations in central, eastern, and southern China in the warm climate zone, Xinjiang is located in an arid climate zone with an annual mean temperature of 10–15 °C and annual precipitation of less than 150 mm, which is not conducive to the formation of AFB1 in feed [26,27,28,29,30]. Moreover, the positive percentage of AFM1 in pasteurized milk in this study in China was less than that found in India (51.3%), Iran (68.0%), and Ethiopia (100%), with lower maximum content than in India (2330 ng/L), Iran (90 ng/L), and Ethiopia (1410 ng/L), and lower mean content than in India (850 ng/L), Iran (33 ng/L), and Ethiopia (970 ng/L) [31,32,33]. Thus, AFM1 contamination of cow milk was relatively much lower in Xiangjiang, China. The large variation in milk AFM1 content in those studies might be related to geographical location, climatic characteristics, feed hygiene conditions, and feeding management.

Consistent with previous studies [14,34], there was no statistical difference in AFM1 contents between pasteurized milk and ESL milk. This may be due to the fact that AFM1 is thermally stable [35].

Raw donkey milk is mainly used in the production of donkey milk powder in Xinjiang, which is a common local livestock product [12]. Similar to previous findings (Table 4), AFM1 was not found in all donkey milk samples (i.e., levels were lower than the detection limit of 5 ng/L) in this study. This indicates a low risk of AFM1 contamination from donkey milk, which may be related to low dietary AFB1 contamination and the low conversion (0.02%) of dietary AFB1 to milk AFM1 [36].

In general, considering the AFM1 contamination of milk from Xinjiang and the strong carcinogenicity of the toxin, it is necessary to assess the current risk of AFM1 exposure for milk consumers in different age groups.

### 4.2. Risk of AFM1 Exposure for Milk Consumers

The AFM1 contamination of milk is widespread in the world [41], and the risk caused by this toxin has attracted much attention. Consistent with those in a previous study in Greece [42], the EDIs and HQs of AFM1 in ten age groups were lower than the hazard limits, indicating that the risk of AFM1 exposure is low for milk consumers in Xinjiang. The low risk for milk consumers may be mainly associated with low milk consumption and low AFM1 content. However, according to the maximum 500 mL of daily milk consumption newly recommended for children [13], combined with the content of milk AFM1 in this study, the 75% percentiles of children’s EDI values were 0.244 (male) and 0.255 (female) ng/kg bw/d for the children aged 2–4 years, while the 95% percentiles were 0.358 (male) and 0.372 (female) ng/kg bw/d for the children aged 2–4 years and 0.252 (male) and 0.263 (female) for 4–7 years, which were higher than the risk limit of 0.2 ng/kg bw/d. Therefore, along with the increase in milk consumption, some children are at risk of exposure, and it is necessary to continuously monitor milk AFM1 levels to strictly control the AFM1 contamination of milk and to ensure the intake of safer dairy products, such as donkey milk. To further reveal the exposure risk, the EDI, HQ, and MOE values for three age groups were reviewed, as shown in Table 5.

Compared with adults and the elderly, children were found to be at higher risk of AFM1 exposure in this study, which was in accordance with findings in previous studies [14,20,21,43]. The higher milk intake per unit of body weight in children than in adults and the elderly may be an important factor for the greater risk of AFM1 exposure. Therefore, to reduce the exposure risk of children, it is necessary to reduce the content of milk AFM1 if milk intake is constant or increased. If the present average daily milk consumption of 151.7 mL in children aged 2–4 years is increased to the maximum 500 mL recommended by CSN (2022) [13], then the mean concentration of AFM1 in milk for children should be controlled to less than 5.64 or 1.61 ng/L, which are the critical concentrations for the HQ or MOE values at risk, respectively.

In this study, the EDIs of AFM1 (0.022–0.053 ng/kg bw/d) for children were far less than 2.910–4.354, 3.30–6.68, 1.01–1.04, and 0.59 ng/kg bw/d in India, Pakistan, Serbia, and Argentina, respectively, and were similar to 0.02–0.17 in Greece (see ref. in Table 5). In addition, the EDI of AFM1 in children in Xinjiang was less than 0.127–0.272 ng/kg bw/d in Shanxi Province [44], China and 0.11–0.25 ng/kg bw/d in central-eastern China [14]. Thus, the risk of AFM1 exposure for children in Xinjiang was lower than that in previous studies. The different EDI values among the above studies compared were affected by AFM1 concentration in milk, daily milk consumption, and consumer body weight, and the difference in AFM1 concentration was a crucial factor.

For the adult group, unlike the EDIs of 0.727–1.089 and 0.78–1.13 ng/kg bw/d in India and Pakistan, respectively, the EDIs of AFM1 for milk consumers in Ghana, Serbia, Greece, China, and Argentina were less than the risk limit. Moreover, the EDI of AFM1 in the elderly group was far less than the risk limit (Table 5). The low risk of AFM1 exposure in these two age groups may be related to their low milk intake per unit of body weight.

The EDI values of AFM1 in three age groups of male and female milk consumers are numerically different (Table 5), which may be related to the sex difference in body weight and milk consumption.

In summary, compared with the elderly and adults, children are at higher risk of AFM1 exposure, and AFM1 contamination of their milk needs to be strictly monitored and controlled under the safe level, considering the susceptibility of children to AFM1.

## 5. Conclusions

We found that 44.4% of cow milk samples in Xinjiang were AFM1-positive in this study, while all raw donkey milk samples were AFM1 negative. The content of AFM1 in cow and donkey milk samples tested was neither higher than the ML in China nor higher than that in the EU. There was no significant difference in content between pasteurized milk and ESL milk. The EDIs of AFM1 in ten age groups were lower than the hazard limits, with the highest EDI of AFM1 in children (2–4 years of age) and the lowest in adults (30–40 years of age). Furthermore, the risk of AFM1 exposure was similar between ten age groups of male and female milk consumers. In summary, the AFM1 contamination of milk in Xinjiang is low and does not represent a serious public health risk, but it is still necessary to continuously monitor it in view of the positive rate of 44.4% and the vulnerability of children to AFM1.

## Figures and Tables

**Table 1:** Occurrence of AFM1 in three types of milk samples from Xinjiang, China.

Milk Type ^a^	Total Sample no.	Positive Sample no. ^b^ (%)	AFM1 Concentration in Positive Samples (ng/L)		
			Concentration Range	Median	Mean ± SD
Pasteurized	93	40 (43.0)	5.1–11.3	7.4	7.4 ± 1.4 ^c^
ESL	96	44 (45.8)	5.1–16.5	7.2	7.8 ± 2.6 ^c^
**Total**	**189**	**84 (44.4)**	**5.1–16.5**	**7.3**	**7.6 ± 2.1**
Donkey milk	70	0	<5	/ ^d^	/

^a^ ESL: extended-shelf-life milk; ^b^ positive samples mean that the milk contains AFM1 at levels > 5 ng/L (the detection of limit). ^c^ Not statistically different (*p* > 0.05). ^d^ “/” indicates that AFM1 levels are unknown, being lower than the 5 ng/L.

**Table 2:** Risk assessment of AFM1 exposure in different age-sex groups of milk consumers.

Age Group	Liquid Milk Consumption (mL/d) ^a^		Body Weight (kg) ^b^		Male EDI ^c^ (ng/kg bw/d)			Female EDI (ng/kg bw/d)		
	Male	Female	Male	Female	Mean	75% Percentile	95% Percentile	Mean	75% Percentile	95% Percentile
2–4	159.6	143.8	14.40	13.80	0.053	0.078	0.114	0.050	0.073	0.108
4–7	135.2	125.2	20.47	19.63	0.032	0.046	0.068	0.030	0.045	0.066
7–11	137.3	136.2	30.38	28.85	0.022	0.032	0.047	0.023	0.033	0.049
11–14	136.2	145.7	43.90	42.30	0.015	0.022	0.032	0.016	0.024	0.036
14–18	136.1	131.4	56.73	50.93	0.011	0.017	0.025	0.012	0.018	0.027
18–30	119.5	121.4	64.83	54.87	0.009	0.013	0.019	0.011	0.016	0.023
30–45	110.9	107.0	68.03	57.73	0.008	0.011	0.017	0.009	0.013	0.019
45–60	118.7	119.1	66.53	59.47	0.009	0.013	0.018	0.010	0.014	0.021
60–70	130	124.4	63.55	57.35	0.010	0.014	0.021	0.010	0.015	0.022
≥70	145.5	139.3	59.67	52.77	0.012	0.017	0.025	0.013	0.019	0.027
**ALL**	**110.9–150.9**	**107.0–145.7**	**14.40–68.03**	**13.80–59.47**	**0.008–0.053**	**0.011–0.078**	**0.017–0.114**	**0.009–0.050**	**0.013–0.073**	**0.019–0.108**

^a^ Zhao and He (2018) [18]. ^b^ Piao and Huo (2019) [17]. ^c^ EDI: estimated daily intake. The concentrations of AFM1-negative milk samples were assumed to be 2.5 ng/L (half of the detection limit of 5 ng/L), from which the mean, 75% percentile, and 95% percentile concentrations were calculated to be 4.78, 7.04, and 10.32 ng/L, respectively. These concentrations were then used to calculate the corresponding EDI values.

**Table 3:** Review of the occurrence of AFM1 in pasteurized milk and ESL milk in China.

Milk Type ^a^	Total Sample no.	Positive Sample no. (%)	Above EU ML ^b^ (>50 ng/L), no. (%)	Above China ML (>500 ng/L), *no*. (%)	Max. Conc. ng/L	Mean ± SD ng/L	Province	Ref.
Pasteurized	26	25 (96.2)	17 (65.4)	0	154	72 ± 41	Beijing and Shanghai	[23]
Pasteurized	410	337 (82.2)	47 (11.5)	0	104.4	27.0 ± 25.0	Hubei, Hunan, and Guangxi	[24]
Pasteurized	288	262 (91.0)	13 (4.5)	0	85.2	18.6 ± 14.8	Hubei, Zhejiang, and Fujian	[14]
**Subtotal**	**724**	**624 (86.2)**	**77 (10.6)**	**0**	**154**	**18.6–72**	**Five provinces + two municipalities**	**Three references**
ESL	16	12 (75.0)	0	0	38	21.3 ± 9.8	Guangdong, Guangxi, Guizhou, and Yunnan	[25]
ESL	93	76 (81.7)	17 (18.3)	0	89.7	28.6 ± 24.1	Hubei, Hunan, and Guangxi	[24]
ESL	120	107 (89.2)	1 (0.8)	0	53.4	16.9 ± 8.8	Hubei, Zhejiang, and Fujian	[14]
**Subtotal**	**229**	**195 (85.2)**	**18 (7.9)**	**0**	**89.7**	**16.9–28.6**	**Eight provinces**	**Three references**
**TOTAL**	**953**	**819 (85.9)**	**95 (10.0)**	**0**	**154**	**16.9–72**	**Eight provinces + two municipalities**	**Four references**

^a^ ESL: extended-shelf-life milk. ^b^ ML: maximum limit.

**Table 4:** Review of the occurrence of AFM1 in donkey milk in previous studies.

Country	Total Sample no.	Positive Sample no. (%)	Max. Conc. ng/L	Mean ± SD ng/L	Ref.
Croatia	14	/ ^a^	10.4	4.77 ± 1.83	[37]
Greece	36	5 (13.9)	26.5	1.60 ± /	[38]
Italy	84	0	/	/	[39]
Italy	63	1 (1.6)	4.4	/	[40]

^a^ Not available in the references.

**Table 5:** Review of the EDIs, HQs, and MOEs of AFM1 in three age groups of male and female milk consumers in previous studies.

Age Group	Gender	EDI ^a^ (ng/kg bw/d)	HQ ^b^ (%)	MOE ^c^	Country	Year	Reference
Children	male	1.04	520.0	548.1	Serbia	2017	[20]
	female	1.01	505.0	564.4	Serbia	2017	[20]
	male	3.30–6.68	1650.0–3340.0	85.3–172.7	Pakistan	2019	[42]
	female	3.48–6.31	1740.0–3155.0	90.3–163.8	Pakistan	2019	[42]
	male	0.127–0.272	63.5–136.0	2095.6–4488.2	China	2020	[44]
	female	0.130–0.255	65.0–127.5	2235.3–4384.6	China	2020	[44]
	both	0.59	295.0	966.1	Argentina	2021	[21]
	male	0.11–0.25	55.0–125.0	2280.0–5181.8	China	2022	[14]
	female	0.11–0.23	55.0–115.0	2478.3–5181.8	China	2022	[14]
	both	0.14–0.45	70.0–225.0	1266.7–4071.4	Ghana	2022	[45]
	both	2.910–4.354	1455.0–2177.0	130.9–195.9	India	2022	[46]
	both	0.02–0.17	10.0–85.0	3352.9–28,500.0	Greece	2022	[43]
	male	0.022–0.053	10.80–26.49	10,754.7–25,909.1	China	2022	This study
	female	0.023–0.050	11.28–24.90	11,400.0–24,782.6	China	2022	This study
**Subtotal**	**male, female, and both**	**0.02–6.68**	**10.0–3340.0**	**85.3–28,500.0**	**Seven countries**	**2017–2022**	**Nine references**
Adult	male	0.082	41.0	6951.2	Serbia	2017	[20]
	female	0.091	45.5	6263.7	Serbia	2017	[20]
	male	0.78	390.0	730.8	Pakistan	2019	[42]
	female	1.13	565.0	504.4	Pakistan	2019	[42]
	male	0.041–0.046	20.5–23.0	12,391.3–13,902.4	China	2020	[44]
	female	0.046–0.055	23.0–27.5	10,363.6–12,391.3	China	2020	[44]
	both	8.69 × 10^−5^	0.04	6,559,263.5	Argentina	2021	[21]
	male	0.04–0.05	20.0–25.0	11,400.0–14,250.0	China	2022	[14]
	female	0.04–0.05	20.0–25.0	11,400.0–14,250.0	China	2022	[14]
	both	0.06–0.19	30.0–95.0	3000.0–9500.0	Ghana	2022	[45]
	both	0.727–1.089	363.5–544.5	523.4–784.0	India	2022	[46]
	both	0.01–0.06	5.0–30.0	9500.0–57,000.0	Greece	2022	[43]
	male	0.008–0.009	3.90–4.41	63,333.3–71,250.0	China	2022	This study
	female	0.009–0.011	4.43–5.29	51,818.2–63,333.3	China	2022	This study
**Subtotal**	**male, female, and both**	**8.69 × 10^−5^–1.13**	**0.04–565.0**	**504.4–6,559,263.5**	**Seven countries**	**2017–2022**	**Nine references**
Elderly	male	0.087	43.5	6551.7	Serbia	2017	[20]
	female	0.117	58.5	4871.8	Serbia	2017	[20]
	male	0.052–0.061	26.0–30.5	9344.3–10,961.5	China	2020	[44]
	female	0.056–0.067	28.0–33.5	8507.5–10,178.6	China	2020	[44]
	male	0.05–0.06	25.0–30.0	9500.0–11,400.0	China	2022	[14]
	female	0.05–0.06	25.0–30.0	9500.0–11,400.0	China	2022	[14]
	both	8.59 × 10^−5^	0.04	6,635,622.8	Argentina	2021	[21]
	both	0.01–0.06	5.0–30.0	9500.0–57,000.0	Greece	2022	[43]
	male	0.010–0.012	4.90–5.83	47,500.0–57,000.0	China	2022	This study
	female	0.010–0.013	5.18–6.31	43,846.2–57,000.0	China	2022	This study
**Subtotal**	**male, female, and both**	**8.59 × 10^−5^–0.117**	**0.04–58.5**	**4871.8–6,635,622.8**	**Four countries**	**2017–2022**	**Six references**
**TOTAL**	**male, female, and both**	**8.59 × 10^−5^–6.68**	**0.04–3340.0**	**85.3–6,635,622.8**	**Seven countries**	**2017–2022**	**Nine references**

^a^ EDI: estimated daily intake. ^b^ HQ: hazard quotient. ^c^ MOE: margin of exposure.

## Data Availability

Data is contained within the article.

## Supplementary Materials

The following supporting information can be downloaded at: https://www.mdpi.com/article/10.3390/foods11233922/s1, Table S1: Content in four types of milk samples.
