# Supplementary material for: Occurrence of Aflatoxin M1 in Three Types of Milk from Xinjiang, China, and the Risk of Exposure for Milk Consumers in Different Age-Sex Groups
Source: Foods. 2022 Dec 5;11(23):3922. doi: 10.3390/foods11233922 (PMC9738243; doi:10.3390/foods11233922)
Supplement: Supplementary file 1 [file foods-11-03922-s001.zip › foods-2030657-supplementary.pdf]

foods

Supplementary materials

Table S1. Content in four types of milk samples.

Sample ID	Milk type	AFM1 concentration (ng/L)	Sample ID	Milk type	AFM1 concentration (ng/L)
B2-1	Pasteurized	9.4	B2-19	Pasteurized	5.6
B2-2	Pasteurized	11.3	B2-20	Pasteurized	6.1
B2-3	Pasteurized	7.2	B2-21	Pasteurized	<LOD
B2-4	Pasteurized	<LOD	B2-22	Pasteurized	6.8
B2-5	Pasteurized	10.5	B2-23	Pasteurized	5.8
B2-6	Pasteurized	<LOD	B2-24	Pasteurized	<LOD
B2-7	Pasteurized	8.7	B2-25	Pasteurized	<LOD
B2-8	Pasteurized	8.3	B2-26	Pasteurized	<LOD
B2-9	Pasteurized	8.3	B2-27	Pasteurized	<LOD
B2-10	Pasteurized	7.6	B2-28	Pasteurized	<LOD
B2-11	Pasteurized	<LOD	B2-29	Pasteurized	6.2
B2-12	Pasteurized	6.5	B2-30	Pasteurized	<LOD
B2-13	Pasteurized	8.5	B2-31	Pasteurized	5.2
B2-14	Pasteurized	8.4	B2-32	Pasteurized	<LOD
B2-15	Pasteurized	9.0	B2-33	Pasteurized	5.1
B2-16	Pasteurized	9.2	B2-34	Pasteurized	5.4
B2-17	Pasteurized	<LOD	B2-35	Pasteurized	<LOD
B2-18	Pasteurized	7.1	B2-36	Pasteurized	7.3
B2-37	Pasteurized	7.7	M3-13	Pasteurized	<LOD
B2-38	Pasteurized	7.5	M3-14	Pasteurized	<LOD
B2-39	Pasteurized	6.2	M3-15	Pasteurized	<LOD
B2-40	Pasteurized	7.6	M3-16	Pasteurized	<LOD
B2-41	Pasteurized	<LOD	M3-17	Pasteurized	<LOD
B2-42	Pasteurized	6.9	M3-18	Pasteurized	<LOD
B2-43	Pasteurized	8.5	M3-19	Pasteurized	<LOD
B2-44	Pasteurized	8.8	M3-20	Pasteurized	<LOD
B2-45	Pasteurized	7.2	M3-21	Pasteurized	<LOD
B2-46	Pasteurized	6.7	M3-22	Pasteurized	<LOD
B2-47	Pasteurized	6.4	M3-23	Pasteurized	<LOD
B2-48	Pasteurized	7.7	M3-24	Pasteurized	<LOD
B2-49	Pasteurized	8.1	M3-25	Pasteurized	<LOD
B2-50	Pasteurized	6.1	M3-26	Pasteurized	<LOD
B2-51	Pasteurized	<LOD	M3-27	Pasteurized	<LOD
B2-52	Pasteurized	5.5	M3-28	Pasteurized	<LOD
B2-53	Pasteurized	<LOD	M3-29	Pasteurized	<LOD
B2-54	Pasteurized	<LOD	A28-1	ESL	<LOD
B2-55	Pasteurized	7.4	A28-2	ESL	5.6
B2-56	Pasteurized	<LOD	A28-3	ESL	<LOD
B2-57	Pasteurized	6.5	A28-4	ESL	<LOD
B2-58	Pasteurized	8.3	A28-5	ESL	5.3
B2-59	Pasteurized	<LOD	A28-6	ESL	<LOD
B2-60	Pasteurized	<LOD	A28-7	ESL	7.3
B2-61	Pasteurized	<LOD	A28-8	ESL	<LOD
B2-62	Pasteurized	<LOD	A28-9	ESL	<LOD
B2-63	Pasteurized	<LOD	A28-10	ESL	7.9
B2-64	Pasteurized	<LOD	A28-11	ESL	5.1
M3-1	Pasteurized	<LOD	A28-12	ESL	<LOD
M3-2	Pasteurized	<LOD	F45-1	ESL	12.5
M3-3	Pasteurized	<LOD	F45-2	ESL	8.3
M3-4	Pasteurized	<LOD	F45-3	ESL	<LOD
M3-5	Pasteurized	<LOD	F45-4	ESL	7.3
M3-6	Pasteurized	<LOD	F45-5	ESL	<LOD
M3-7	Pasteurized	<LOD	F45-6	ESL	7.0
M3-8	Pasteurized	<LOD	F45-7	ESL	<LOD

Sample ID	Milk type	AFM1 concentration (ng/L)	Sample ID	Milk type	AFM1 concentration (ng/L)
M3-9	Pasteurized	<LOD	F45-8	ESL	<LOD
M3-10	Pasteurized	<LOD	G28-1	ESL	<LOD
M3-11	Pasteurized	<LOD	G28-2	ESL	<LOD
M3-12	Pasteurized	<LOD	G28-3	ESL	8.9
G28-4	ESL	5.7	C45-10	ESL	8.5
G28-5	ESL	<LOD	D45-1	ESL	11.2
G28-6	ESL	<LOD	D45-2	ESL	10.8
G28-7	ESL	5.2	D45-3	ESL	16.5
G28-8	ESL	5.8	D45-4	ESL	7.6
G28-9	ESL	7.2	D45-5	ESL	8.8
G28-10	ESL	6.8	D45-6	ESL	<LOD
G28-11	ESL	<LOD	D45-7	ESL	5.4
H28-1	ESL	<LOD	D45-8	ESL	<LOD
H28-2	ESL	6.8	D45-9	ESL	<LOD
H28-3	ESL	<LOD	E45-1	ESL	11.4
H28-4	ESL	6.7	E45-2	ESL	9.8
H28-5	ESL	5.7	E45-3	ESL	6.3
H28-6	ESL	<LOD	E45-4	ESL	<LOD
H28-7	ESL	<LOD	E45-5	ESL	5.8
H28-8	ESL	<LOD	E45-6	ESL	<LOD
H28-9	ESL	<LOD	E45-7	ESL	<LOD
H28-10	ESL	<LOD	E45-8	ESL	13.9
I21-1	ESL	6.3	E45-9	ESL	<LOD
I21-2	ESL	7.8	E45-10	ESL	<LOD
I21-3	ESL	5.2	E45-11	ESL	<LOD
I21-4	ESL	<LOD	K45-1	ESL	10.9
I21-5	ESL	<LOD	K45-2	ESL	<LOD
I21-6	ESL	<LOD	K45-3	ESL	<LOD
I21-7	ESL	5.3	K45-4	ESL	<LOD
I21-8	ESL	<LOD	K45-5	ESL	5.4
J45-1	ESL	10.8	K45-6	ESL	<LOD
J45-2	ESL	5.1	K45-7	ESL	<LOD
J45-3	ESL	<LOD	K45-8	ESL	<LOD
J45-4	ESL	<LOD	L45-1	ESL	8.5
J45-5	ESL	5.7	L45-2	ESL	8.4
C45-1	ESL	8.3	L45-3	ESL	<LOD
C45-2	ESL	<LOD	L45-4	ESL	<LOD
C45-3	ESL	10.1	N1-1	Donkey milk	<LOD
C45-4	ESL	<LOD	N1-2	Donkey milk	<LOD
C45-5	ESL	5.6	N1-3	Donkey milk	<LOD
C45-6	ESL	<LOD	N1-4	Donkey milk	<LOD
C45-7	ESL	<LOD	N1-5	Donkey milk	<LOD
C45-8	ESL	<LOD	N1-6	Donkey milk	<LOD
C45-9	ESL	<LOD	N1-7	Donkey milk	<LOD
N1-8	Donkey milk	<LOD	O1-8	Donkey milk	<LOD
N1-9	Donkey milk	<LOD	O1-9	Donkey milk	<LOD
N1-10	Donkey milk	<LOD	O1-10	Donkey milk	<LOD
N1-11	Donkey milk	<LOD	O1-11	Donkey milk	<LOD
N1-12	Donkey milk	<LOD	O1-12	Donkey milk	<LOD
N1-13	Donkey milk	<LOD	O1-13	Donkey milk	<LOD
N1-14	Donkey milk	<LOD	O1-14	Donkey milk	<LOD
N1-15	Donkey milk	<LOD	O1-15	Donkey milk	<LOD
N1-16	Donkey milk	<LOD	O1-16	Donkey milk	<LOD
N1-17	Donkey milk	<LOD	O1-17	Donkey milk	<LOD
N1-18	Donkey milk	<LOD	O1-18	Donkey milk	<LOD
N1-19	Donkey milk	<LOD	O1-19	Donkey milk	<LOD
N1-20	Donkey milk	<LOD	O1-20	Donkey milk	<LOD
N1-21	Donkey milk	<LOD	O1-21	Donkey milk	<LOD
N1-22	Donkey milk	<LOD	O1-22	Donkey milk	<LOD

Sample ID	Milk type	AFM1 concentration (ng/L)	Sample ID	Milk type	AFM1 concentration (ng/L)
N1-23	Donkey milk	<LOD	O1-23	Donkey milk	<LOD
N1-24	Donkey milk	<LOD	O1-24	Donkey milk	<LOD
N1-25	Donkey milk	<LOD	O1-25	Donkey milk	<LOD
N1-26	Donkey milk	<LOD	O1-26	Donkey milk	<LOD
N1-27	Donkey milk	<LOD	O1-27	Donkey milk	<LOD
N1-28	Donkey milk	<LOD	O1-28	Donkey milk	<LOD
N1-29	Donkey milk	<LOD	O1-29	Donkey milk	<LOD
N1-30	Donkey milk	<LOD	O1-30	Donkey milk	<LOD
N1-31	Donkey milk	<LOD	O1-31	Donkey milk	<LOD
N1-32	Donkey milk	<LOD	O1-32	Donkey milk	<LOD
O1-1	Donkey milk	<LOD	O1-33	Donkey milk	<LOD
O1-2	Donkey milk	<LOD	O1-34	Donkey milk	<LOD
O1-3	Donkey milk	<LOD	O1-35	Donkey milk	<LOD
O1-4	Donkey milk	<LOD	O1-36	Donkey milk	<LOD
O1-5	Donkey milk	<LOD	O1-37	Donkey milk	<LOD
O1-6	Donkey milk	<LOD	O1-38	Donkey milk	<LOD
O1-7	Donkey milk	<LOD			

Note: (1) Sample ID: the first one or two characters indicate the manufacturer, followed by numbers showing the story life (days). And then the last one to two numbers after "dash" are the sampling number. (2) ESL: **e**xtended **s**helf **l**ife milk; UHT: **u**ltra-**h**igh **t**emperature milk. (3) LOD = 5 ng/L.
